# 5^th^ International AIDS Society Conference on HIV Pathogenesis, Treatment and Prevention: summary of key research and implications for policy and practice - Introduction

**DOI:** 10.1186/1758-2652-13-S1-S1

**Published:** 2010-06-01

**Authors:** Rodney Kort

**Affiliations:** 1 Kort Consulting, Toronto, M4Y 2T6, Canada

## Introduction

Attracting more than 5800 participants from 123 countries, the 5^th^ IAS Conference on HIV Pathogenesis, Treatment and Prevention (IAS 2009), held in Cape Town, South Africa, on 19-23 July 2009, offered more than 1550 reports on original research in four areas: basic sciences (Track A), clinical sciences (Track B), biomedical prevention (Track C), and – new at this meeting – operations research (Track D). The 59 sessions included 12 plenary addresses by leaders from every area of HIV research. The conference was particularly rich in research that may affect the path of the epidemic in low- and middle-income countries, although policy- and practice-shaping developments from high-income countries were also present.

Consistent with the focus of the pathogenesis conference series, this report aims not only to highlight particularly important original research and other developments from IAS 2009, but also to analyze their potential impact on policy and practice in the coming years for those working in HIV and related fields. The report includes abstract numbers of cited presentations linked to online files of slides, posters or abstracts, as available. A full abstract search of IAS 2009 and prior IAS conferences is available on the IAS website at http://www.iasociety.org as well as on the JIAS website at http://www.jiasociety.org/.

New data presented at IAS 2009 is already having an impact on HIV policy and practice on a global scale.

■ Results of several basic research studies provided the field with a better understanding of the elevated HIV infection risk among African women compared with women from the USA due to chronically activated T cells and higher levels of cytokines favouring HIV transmission in genital tract mucosa (likely a result of increased exposure to pathogens), how complex genetic variables may affect HIV acquisition and disease progression, and how early antiretroviral therapy (ART) can substantially reduce the size of latent HIV reservoirs, a significant clinical issue in chronic HIV infection.

■ Data from a study demonstrating that pre-ART inflammation and coagulation markers predict death and related findings showing that HIV replication induces activation of tissue factor pathways, thrombosis and fibrinolysis (with associated increases in mortality) underscored the growing recognition of how HIV permits chronic inflammation as a paradigm shift in our understanding of HIV disease progression.

■ Findings demonstrating that maternal triple-drug ART used throughout pregnancy and breastfeeding reduced vertical transmission to 1% (and lowered the risk of prematurity, stillbirth and abortion) are expected to inform revised World Health Organization (WHO) and South African national guidelines on antiretroviral prophylaxis.

■ Research delineating the impact of antiretroviral therapy on reducing coincident tuberculosis and malaria epidemics in HIV-prevalent regions argued for wider and earlier access to treatment. By the end of the conference, South African health authorities indicated they would consider providing ART to everyone co-infected with TB and HIV.

■ In the biomedical prevention field, new work helped define the impact of pregnancy on HIV transmission in serodiscordant couples and the apparent lack of a direct effect of male circumcision on HIV acquisition in female partners.

■ An updated presentation of a WHO modelling study, which suggests that universal voluntary testing and immediate ART could turn the tide of the epidemic, was bolstered by additional data from recent studies demonstrating the potential preventive impact of ART at a population level. The significant challenges of operationalizing such a strategy remain to be elaborated.

■ A study indicating that early ART intervention for everyone with a CD4+ count of less than 350 cells/mm^3^ was more cost effective than deferred ART intervention added to the growing scientific consensus that normative guidance should advise earlier ART initiation, particularly given the recently suspended Comprehensive International Program of Research on AIDS (CIPRA) HT 001 trial in Haiti, which found substantially higher rates of mortality and morbidity in the deferred versus early ART intervention arms.

■ Operations research presented at the conference provided important new insights into how integrating HIV with other health services and using a variety of service delivery approaches (including deploying trained community or lay workers) can exponentially expand health system capacity without compromising standards of care or treatment outcomes.

■ Results from the five-year Development of Anti-Retroviral Therapy in Africa (DART) trial comparing laboratory CD4+ and viral load monitoring with clinical monitoring found minimal differences in virologic outcomes, but substantially higher costs associated with laboratory monitoring. These and other data from IAS 2009 suggest that CD4+ count monitoring is cost effective as a targeted, rather than routine, strategy.

■ A provocative overview of AIDS financing suggested that much more could be done to get “less AIDS for the money” by ensuring that interventions are evaluated more consistently for efficacy and cost effectiveness, and strategically targeted for maximum benefit given the widely varying epidemics in different regions.

## Methods

The International AIDS Society (IAS) recruited writers with content expertise in the four conference tracks to draft a post-conference report. Throughout the conference and in the closing session, four teams of rapporteurs summarized all oral and many poster presentations, and discussed their relevance in the context of research and the provision of care, treatment and provision. This report is based on a review of all individual and summary rapporteur reports back, additional conference sessions and satellites worthy of note. It also draws upon important findings from additional studies in recently published scientific literature and, where relevant, media reports.

Each writer then submitted reports on specific tracks which were reviewed, edited and combined into a draft report. The Impact Report: Summary of Key Research and Implications for Policy and Practice – 5^th^ International AIDS Society Conference on HIV Pathogenesis, Treatment and Prevention was released in October 2009 and is available online at http://www.iasociety.org. That report was revised to meet the author specifications for publication as a supplement of the *Journal of the International AIDS Society*, and peer reviewed prior to publication.

## Discussion

As the first IAS conference to return to sub-Saharan Africa since the watershed XIII International AIDS Conference (AIDS 2000) in Durban, IAS 2009 served to highlight both the enormous progress in HIV treatment, prevention and care over the past decade, and the ongoing challenges of delivering HIV interventions in a resource-limited region that remains the epicentre of the global epidemic. This is the last conference of the pathogenesis series before the 2010 deadline for universal access, set by the G8 and subsequently by the international community at the World Summit in 2005.

The research, discussions and debate at IAS 2009 demonstrated both the necessity of establishing that ambitious goal, and the growing concern that despite the extraordinary progress in sub-Saharan Africa and most other regions on country-level targets for 2010, it is unlikely that many countries will meet their 2010 goal of universal access to a package of care, treatment and support. This is due to a mix of shifting political priorities, inadequate resources, the refusal of some countries to implement evidence-based interventions, the recent global economic crisis, and the enormous challenges in delivering these interventions, based on fundamental human rights principles, in health care systems that are chronically under resourced.

The focus in this supplement is on profiling the presentations, discussions and debates that are likely to have the most significant impact on the epidemic in advancing an evidence-based response to HIV/AIDS.

The purpose of the IAS 2009 Impact Report and this *Journal of the International AIDS Society* supplement is to advance the response to AIDS by profiling important new research and emerging discussions and translating them into the evidence-based policies and programmes required to achieve universal access to HIV prevention, treatment, care and support.

In any conference as large and diverse as IAS 2009, new findings and worthy research are presented on a wide range of topics. To ensure a manageable length for the report (and, ultimately, to expand its readership), we have limited the number of studies referenced in this report to those that demonstrated the most substantive potential impact on future research, policy development (including normative guidance) and related clinical and health care delivery interventions. A complete source of all conference abstracts is available online at either the JIAS Abstract Database (http://www.jiasociety.org/) or the IAS Abstract Database (http://www.iasociety.org).

### Organization of the supplement

This supplement is divided into an Introduction and four subsequent sections (one covering each of the four conference tracks), each of which includes an analysis of the implications and potential impact of the major developments reported at IAS 2009 in the areas of research, programme development, policy and advocacy. The four subsequent sections are:

**2. Basic sciences:** An analysis of new evidence presented in Track A, as well as in related sessions, activities and affiliated events.

**3. Clinical sciences:** An analysis of new evidence presented in Track B, as well as in related sessions, activities and affiliated events.

**4. Biomedical prevention:** An analysis of new evidence presented in Track C, as well as in related sessions, activities and affiliated events.

**5. Operations research:** An analysis of new evidence presented in this inaugural track (Track D) of the pathogenesis conference, including related sessions, activities and affiliated events. A clear and widely accepted definition of operations research continues to evolve, but encompasses a wide range of disciplines using research (including mathematical modelling and cost-effectiveness studies) to inform the evaluation and delivery of HIV prevention, care and treatment interventions.

## Conclusions

One of the most important takeaway messages from the conference is the wealth of new data that is expected to inform upcoming WHO and national clinical guidance on ART treatment for adults, adolescents and paediatrics, and the significant impact this will have, not only on people living with HIV, but also for people at risk for or living with tuberculosis or malaria; these three diseases continue to be the world’s three largest causes of mortality.

Research on the impact of ART on pregnancy outcomes is also likely to inform guidance for HIV-positive pregnant and lactating women. For example, WHO released update guidance in November 2009, recommending prolonged use of antiretrovirals (ARVs) to reduce the risk of mother to child transmission of HIV and, for the first time, that HIV-positive mothers or their infants take ARVs while breastfeeding to prevent HIV transmission. Also, data from IAS 2009 expanded our understanding of circumcision and its limits. A number of important studies in Track D will inform discussions of the (expanded) role of laboratory services, including the key role that simple, point-of-care tests could play in diagnosing and treating HIV early in disease progression, and also on the need to more frequently evaluate all HIV programmes and service delivery modalities for their effectiveness and to ensure that programmes are context specific, evidence based and designed to maximize their impact on local treatment, care and epidemiology.

IAS 2009 will bring renewed evidence of the impact of HIV policies and programmes to political leaders in the lead up to the 2010 G8 Summit in Canada, AIDS 2010 in Vienna and the 2010 United Nations General Assembly Special Session (UNGASS) on HIV/AIDS in New York. It also brings with this evidence the caution that the success to date is fragile and underfunded, dependent on countries meeting their international and national commitments with the best available scientific evidence, and dependent on ensuring that the response to AIDS remains a global priority beyond 2010.

The JIAS hopes that this supplement will be a useful resource for HIV professionals working in every sector of the response to AIDS, and that it will become a powerful evidence-based advocacy tool used to strengthen and expand the response to HIV/AIDS worldwide.

## Competing interests

Rodney Kort is an independent consultant contracted by the International AIDS Society for the purpose of preparing the IAS 2009 Impact Report (Executive Summary, Introduction and Track D coverage) and adapting the entire report for publication in the *Journal of the International AIDS Society*.

## Author’s contributions

RK drafted this text and approved the manuscript for publication.

